# Time- and temperature-dependent postmortem concentration changes of the (synthetic) cannabinoids JWH-210, RCS-4, as well as ∆9-tetrahydrocannabinol following pulmonary administration to pigs

**DOI:** 10.1007/s00204-020-02707-4

**Published:** 2020-03-18

**Authors:** Nadine Schaefer, Ann-Katrin Kröll, Christina Körbel, Matthias W. Laschke, Michael D. Menger, Hans H. Maurer, Markus R. Meyer, Peter H. Schmidt

**Affiliations:** 1 Institute of Legal Medicine, Saarland University, Building 49.1, 66421 Homburg, Germany; 2 Institute for Clinical and Experimental Surgery, Saarland University, Building 65/66, 66421 Homburg, Germany; 3 Department of Experimental and Clinical Toxicology, Center for Molecular Signaling (PZMS), Saarland University, Building 46, 66421 Homburg, Germany

**Keywords:** Synthetic cannabinoids, Tetrahydrocannabinol, Postmortem redistribution, Pigs, Pulmonary administration

## Abstract

In forensic toxicology, interpretation of postmortem (PM) drug concentrations might be complicated due to the lack of data concerning drug stability or PM redistribution (PMR). Regarding synthetic cannabinoids (SC), only sparse data are available, which derived from single case reports without any knowledge of dose and time of consumption. Thus, a controlled pig toxicokinetic study allowing for examination of PMR of SC was performed. Twelve pigs received a pulmonary dose of 200 µg/kg BW each of 4-ethylnaphthalene-1-yl-(1-pentylindole-3-yl)methanone (JWH-210), 2-(4-methoxyphenyl)-1-(1-pentyl-indole-3-yl)methanone (RCS-4), and Δ9-tetrahydrocannabinol via an ultrasonic nebulizer. Eight hours after, the pigs were put to death with T61 and specimens of relevant tissues and body fluids were collected. Subsequently, the animals were stored at room temperature (*n* = 6) or 4 °C (*n* = 6) and further samples were collected after 24, 48, and 72 h each. Concentrations were determined following enzymatic cleavage and solid-phase extraction by liquid-chromatography tandem mass spectrometry applying the standard addition approach. High concentrations of the parent compounds were observed in lung, liver, kidney and bile fluid/duodenum content as well as brain. HO-RCS-4 was the most prevalent metabolite detected in PM specimens. In general, changes of PM concentrations were found in every tissue and body fluid depending on the PM interval as well as storage temperature.

## Introduction

In general, the interpretation of postmortem (PM) data is challenging for a forensic toxicologist, because in most of the cases, dose and time of intake are unknown. Usually, the survival time after intake, the time of death and the PM interval (PMI) are also unknown. However, those parameters are very important regarding the interpretation of a drug concentration, especially in the context of a possible overdose. It has to be considered that a concentration calculated in a PM specimen does not necessarily reflect the peak concentration during lifetime and not even at the time of death. For this reason, reference lists of therapeutic concentration ranges have to be utilized with caution. During the agony until death occurs, a substance might be further metabolized and excreted into urine. Furthermore, an increasing PMI might lead to degradation of a substance due to instability or further metabolism via microorganisms in the corpse or the environment (Martínez-Ramírez et al. 2016; Robertson 1995). Those phenomena are summarized in the term PMR. Further mechanisms can contribute to PMR and alter postmortem drug concentrations. Depending on the physicochemical and toxicokinetic (TK) properties (i.e. volume of distribution, lipid solubility of a drug) concentrations can change because of diffusion processes or pH changes in different tissues (Sastre et al. 2017; Pélissier-Alicot et al. 2003; Skopp 2010). Furthermore, drugs might be released from several organs acting as drug reservoir and diffuse to sites of low drug concentration (Sastre et al. 2017; Pélissier-Alicot et al. 2003; Skopp 2010). When interpreting concentrations in PM blood specimens collected from different sites of the body [i.e. central versus peripheral blood (CB/PB)], such issues have to be considered (Crandall et al. 2006; Flanagan et al. 2003; Zilg et al. 2017).

PM cases involving new psychoactive substances (NPS) entail several further difficulties, as usually no data are available concerning their TK and toxicodynamic properties, especially in terms of potency and toxicity. However, the NPS, especially the synthetic cannabinoids (SC) have gained increasing popularity both in local media and media worldwide. Up to now, a lot of fatalities with SC at least contributing to the occurrence of death have already been reported (Boland et al. 2019; Darke et al. 2019; Kraemer et al. 2019; Yamagishi et al. 2018). Besides these single case reports, some systematic and few controlled animal studies have been performed to obtain information about the tissue distribution of SC (Castaneto et al. 2015; Meyer 2016). However, these studies did not provide comprehensive information on the tissue distribution of SC, as only certain organs and/or blood were analyzed, and the distribution of their metabolites has not been examined. Regarding this issue, a pig model suitable for cannabinoid TK studies after pulmonary administration of 4-ethylnaphthalene-1-yl-(1-pentylindole-3-yl)methanone (JWH-210), 2-(4-methoxyphenyl)-1-(1-pentyl-indole-3-yl)methanone (RCS-4), and tetrahydrocannabinol (THC) has been established by the authors providing antemortem data on the distribution of SC and their main metabolites in blood (Schaefer et al. 2018a, b) as well as perimortem data on the distribution in the different organs at the time of death (Schaefer et al. 2019). Pigs were chosen, because they have already been proven to be useful examining postmortem concentration changes of central nervous acting substances (Brunet et al. 2010; Crandall et al. 2006; Flanagan et al. 2003; Hilberg et al. 1998).

Following, the PM distribution as well as the time- and temperature-dependent concentration changes of the cannabinoids and their main metabolites should be examined in the present study and results should be compared with those found in the perimortem specimens (Schaefer et al. 2019).

## Materials and methods

### Chemicals and reagents

Acetone Supra Solv, methanol Supra Solv, glacial acetic acid p.a., formic acid, sodium bicarbonate, potassium hydroxide, di-potassium hydrogen phosphate, β-glucuronidase/arylsulfatase (from Helix pomatia), and HPLC grade water were purchased from VWR-International (Darmstadt, Germany). Ethanol p.a. and HPLC grade acetonitrile were obtained from Sigma-Aldrich (Steinheim, Germany). Methanolic solutions of THC (0.1 mg/mL), THC pharmaceutical grade for drug administration (Dronabinol, DAC 2008, 98.5% purity), JWH-210 (solid), and RCS-4 (solid) were purchased from THC Pharm (Frankfurt/Main, Germany), THC-d3, 11-hydroxy-THC (HO-THC), HO-THC-d3, 11-nor-9-carboxy-THC (THC-COOH), and THC-COOH-d3 solution (0.1 mg/mL each) from LGC/Promochem (Wesel, Germany), and methanolic solutions of JWH-210-d9 (1 mg/mL) and RCS-4-d9 (5 mg/mL), hydroxypentyl-RCS-4 (HO-RCS-4) solution (10 mg/mL in acetonitrile), hydroxypentyl-JWH-210 (HO-JWH-210, solid), JWH-210-pentanoic acid (JWH-210-COOH, solid), and RCS-4-pentanoic acid (RCS-4-COOH, solid) from Cayman Europe (Tallinn, Estonia). JWH-210 for drug administration was provided by the German Federal Criminal Police Office (Wiesbaden, Germany) and RCS-4 (96% purity) was purchased as ‘research chemical’ from an internet provider.

The buffers were prepared as described elsewhere (Schaefer et al. 2015, 2019). The acetate buffer (pH 4) was prepared with 5.7 mL of glacial acetic acid and 16 mL of aqueous potassium hydroxide (1 M). For the preparation of the sodium bicarbonate solution, 50 g sodium bicarbonate was dissolved in 1 L deionized water. The phosphate buffer (0.1 M, pH 9) was prepared by dissolving 22.82 g di-potassium hydrogen phosphate in 1 L deionized water.

### Blank whole blood specimens

Blank blood specimens used for the preparation of calibrators and quality controls were obtained from drug-free pigs.

### Stock solutions, calibration standards, and quality control samples

Standard stock solutions containing the concentration of 1 mg/mL were prepared by dissolving 5 mg of each solid compound in 5 mL ethanol. The stock solutions or liquid reference standards were then diluted to obtain working standard solutions (0.001, 0.01, 0.1 mg/mL). Calibrator standard spiking solutions were created in whole blood in concentrations of 0.5, 1, 2, 6, 10, 20, 30, and 50 ng/mL by diluting working solutions with ethanol. Quality control LOW (1.5 ng/mL for JWH-210 and metabolites, RCS-4 and HO-RCS-4; 5.0 ng/mL for RCS-4-COOH and THC and metabolites) and HIGH (15 ng/mL for JWH-210 and metabolites and RCS-4 and metabolites; 25 ng/mL for THC and metabolites) specimens were also prepared by diluting working solutions with ethanol (Schaefer et al. 2015). All solutions were stored at − 20 °C.

### Calibrators for standard addition

As already described in a previous study (Schaefer et al. 2019), standard stock solutions (1 mg/mL) were prepared by dissolving 5 mg of each solid compound in 5 mL of ethanol. Concentrations of working standard solutions (0.001 mg/mL, 0.01 mg/mL, 0.1 mg/mL) were obtained by diluting stock solution or liquid reference standards with ethanol, respectively. The concentrations of the calibrators used for standard addition were 20, 40, and 60 ng/g or ng/mL. All solutions were stored at − 20 °C.

### Animals

As described elsewhere (Schaefer et al. 2019), twelve domestic male pigs [Swabian Hall strain; body weight (BW) 40.5–49.8 kg] were used for the study. The animals had free access to tap water and daily standard chow. They were kept fasting a night before the experiment with free access to water.

### Surgical procedures

Surgical procedures have already been described elsewhere (Schaefer et al. 2015, 2016, 2017a, b, 2018a, b, 2019). In brief, ketamine hydrochloride (30 mg/kg, Ursotamin; Serumwerk Bernburg, Bernburg, Germany), xylazine hydrochloride (2.5 mg/kg, Rompun; Bayer, Leverkusen, Germany), and atropine (1 mg, Braun, Melsungen, Germany) were injected for premedication intramusculary. Analgosedation was obtained by isoflurane (2–4%, Forene, AbbVie, Ludwigshafen, Germany). Pigs were mechanically ventilated with a mixture of oxygen and air (1:2 vol/vol; FiO_2_ of 0.30; Respirator ABV-U; F. Stephan GmbH, Gackenbach, Germany) and volume cycled with a tidal volume of 10–12 mL/kg. The jugular vein was catheterized with a triple-lumen 7F (Certofix Trio, Braun, Melsungen, Germany) central venous catheter for monitoring of mean central venous pressure. The left ear vein was catheterized for fluid replacement [sodium chloride 0.9% (8 mL kg^−1^ h^−1^), Braun, Melsungen, Germany]. Additionally, a catheter (Leadercath Expert 14G, Vygon, Aachen, Germany) was inserted into the left femoral artery for invasive blood pressure measurement and blood gas analysis. Finally, a suprapubic catheter (Cystofix, Braun, Melsungen, Germany) was placed into the bladder for urine sample collection. The animals were then allowed to stabilize for 10–15 min.

### Study design

As already described elsewhere (Schaefer et al. 2019; Schaefer et al. 2018a, b), a stock solution of 7.5 mg/mL of JWH-210, RCS-4, and THC each was first prepared in ethanol. The appropriate volume of the solution (1080–1328 µL) was applied to obtain a 200 µg/kg BW dose, respectively. The dose was administered within 12 min by nebulization of the drugs applying the inspiration-triggered mode (< 0.2 mL/min) of the M-neb flow^+^ ventilation ultrasonic nebulizer MN-300/7 (Nebutec, Elsenfeld, Germany). The administration set-up has already been described in detail in a previous study (Schaefer et al. 2018).

As described in a previous study (Schaefer et al. 2019), eight hours after administration (PMI 0), the animals were euthanized with T 61 (0.12 mL/kg BW, Intervet Deutschland GmbH, Unterschleißheim, Germany) and the abdominal cavity was opened. Subsequently, biopsies of the following organs and tissues, as well as body fluids were collected by leaving the organs in situ: lung, liver, kidney, skeletal muscle tissue, peripheral blood from the jugular vein (PB), central blood (CB), bile fluid as well as duodenum content. Additionally, specimens of the brain (cerebrum, cerebellum) were sampled.

Afterwards, the abdominal cavity was sutured, the animals were stored at room temperature (RT; *n* = 6) or 4 °C (*n* = 6) in a supine position and further specimens were collected after 24, 48, and 72 h (PMI 1–3) each as described above with one exception. The PB PM blood at PMI 1–3 was collected from the femoral or the brachiocephalic vein. All samples were stored at − 20 °C until analysis.

### Sample preparation

#### Blood specimens

Specimens were prepared according to a fully validated method in each described elsewhere (Schaefer et al. 2015, 2019). Limits of detection were 0.05 ng/mL for JWH-210 and JWH-210-COOH, 0.10 ng/mL for HO-JWH-210, 0.15 ng/mL for RCS-4 and HO-RCS-4 and 0.5 ng/ml for RCS-4-COOH, THC, HO-THC and THC-COOH. Lower limits of quantification were assessed at 0.5 ng/mL for JWH-210, HO-JWH-210, JWH-210-COOH, RCS-4 and HO-RCS-4 as well as 2.0 ng/mL for RCS-4-COOH, THC, HO-THC and THC-COOH (Schaefer et al. 2015).

In brief, a solid phase extraction (SPE) was performed using Strata C18 endcapped cartridges (200 mg/3 mL; Phenomenex LTD, Aschaffenburg, Germany). The columns were conditioned with 2 × 3 mL methanol and 3 mL phosphate buffer (0.1 M, pH 9). Whole blood specimens were homogenized and an aliquot of 250 µL was added to a mixture of 20 µL of an ethanolic stable-isotope-labeled internal standard mixture solution (SIL-IS, 2 ng/20 µL of JWH-210-d9 and RCS-4-d9, 10 ng/20 µL of THC-d3, HO-THC-d3, and THC-COOH-d3), 25 µL ethanol, and 2.75 mL phosphate buffer. For preparation of calibrators and quality controls 25 µL of ethanol were replaced by the appropriate spiking solution. The specimens were then vortexed, centrifuged and subsequently loaded onto cartridges. Three washing steps with 3 mL phosphate buffer, 3 mL acetic acid (0.25 M), and 3 mL water followed and the columns were dried using 10 in. Hg after adding 60 µL acetone. Analytes were eluted by adding 1.5 mL methanol–acetone (1:1, v/v). Following, the eluates were evaporated under nitrogen, and the dry residues dissolved in 100 µL of a mixture of mobile phases A and B (50:50, v/v). Mobile phase A consisted of 0.1% aqueous formic acid and B was 0.1% formic acid in acetonitrile. Twenty microliters were then injected onto the liquid-chromatography tandem mass spectrometry (LC–MS/MS) system.

#### Tissue specimens

As already described in a previous study (Schaefer et al. 2017b, 2019), 2 g solid tissue (brain, lung, liver, kidney, and muscle tissue) or bile fluid and duodenum content was homogenized (1 amount tissue/bile fluid/duodenum content + 4 amounts water). Subsequently, four 0.5-g portions were prepared with and without addition of different concentrations of JWH-210, HO-JWH-210, JWH-210-COOH, RCS-4, HO-RCS-4, RCS-4-COOH, THC, 11-HO-THC, and THC-COOH to create a standard addition calibration curve. Following, 20 µL IS was added together with 500 µL of acetate buffer and 50 µL β-glucuronidase/arylsulfatase. The mixture was vortexed and incubated for 2 h at 60 °C for hydrolysis. The specimens were then fortified with 1 mL acetonitrile, vortexed, and centrifuged at 3500*g* for 8 min. An amount of 1 mL sodium bicarbonate solution was added to the supernatants and the mixture was vortexed. Subsequently, SPE was carried out as described above. Twenty microliters was then injected onto the LC–MS/MS system.

### Standard addition method

In the current study, the standard addition approach was applied to quantify the drugs and their metabolites in tissues and bile fluid/duodenum content. Four portions were prepared of each specimen, one containing no calibrator solution and three containing different concentrations of calibrator solution. Regression analysis was then performed by creating standard addition calibration equations as follows: *y* = *ax* + *b*. Depending on the slope (*a*) and the intercept (*b*) the calibration curve intersects the *x*-axis at the negative side. The point of intersection represents the unknown concentration.

### Apparatus

#### LC–MS/MS

LC–MS/MS conditions including instrumentation, chromatographic, and mass spectrometric conditions for the analysis of extracts have already been described elsewhere (Schaefer et al. 2015, 2017b, 2019). Briefly, a Thermo Fisher (TF, Dreieich, Germany) HPLC consisting of one Allegro pump, and an HTC PAL autosampler was applied. Detection was achieved using a TF TSQ Quantum Ultra Accurate Mass triple stage mass spectrometer with an atmospheric pressure chemical ionization (APCI) interface run in the positive mode. A Waters (Wexford, Ireland) Sunfire C_18_ column (150 × 2.1 mm, 3.5 µm) with a gradient elution was applied using mobile phase A and B. The runtime was about 10 min. Ionization was achieved with the APCI source in positive mode and following settings: discharge current 5.0 µA; vaporizer temperature 400 °C; sheath gas 40 arbitrary units; auxiliary gas 15 arbitrary units; capillary temperature 270 °C. Detection and quantification of the compounds were carried out in multiple-reaction monitoring mode with three transitions per precursor ion. TF Xcalibur Version 2.0.7 SP 1 software was used.

### Calculation of concentrations changes

The median concentration changes of the drugs and their metabolites determined at PMI 1–3 in comparison with the median perimortem concentrations = PMI 0 (see Table 1) were calculated using the following equation:

$$\begin{aligned} & \Delta c(\% ) = \frac{{c({\text{PMI}}\,1 - 3) - c({\text{PMI}}\,0)}}{{c({\text{PMI}}\,0)}} \times 100. \\ & \Delta c > 0{:}\,{\text{increase}} \\ & \Delta c < 0{:}\,{\text{decrease}} \\ \end{aligned}$$

**Table 1:** Median and mean [± standard deviation (SD)] concentrations of JWH-210, RCS-4 and THC in tissues and body fluids measured at postmortem interval (PMI) 0 according to Schaefer et al. (2019) and PMI 1–3 stored at 4 °C or room temperature (RT); concentrations are approximated (except for those marked with asterisk) and displayed as one decimal non-zero

	JWH-210 Median conc. [Mean conc. ± SD] in ng/mL or ng/g							
	RT				4 °C			
	PMI 0	PMI 1	PMI 2	PMI 3	PMI 0	PMI 1	PMI 2	PMI 3
PB	0.5* [0.8 ± 0.7] (*n *= 6)	0.6* [1 ± 2] (*n *= 4)	0.1 [0.3 ± 0.4] (*n *= 4)	1* [1 ± 0.7] (*n *= 4)	1* [1 ± 0.6] (*n *= 3)	0.3 [0.5 ± 0.4] (*n *= 4)	0.7* [1 ± 1] (*n *= 4)	1* [1 ± 0.6] (*n *= 4)
CB	1 [1 ± 0.5] (*n *= 4)	1* [2 ± 2] (*n *= 6)	1* [1 ± 0.7] (*n *= 6)	1* [1 ± 0.7] (*n *= 6)	0.2 [0.7 ± 0.3] (*n *= 4)	0.9* [0.7 ± 0.3] (*n *= 4)	0.7* [0.6 ± 0.2] (*n *= 4)	0.4 [0.4 ± 0.3] (*n *= 4)
Cerebrum	8 [11 ± 7] (*n *= 6)	8 [11 ± 7] (*n *= 6)	30 [34 ± 18] (*n *= 6)	36 [39 ± 26] (*n *= 6)	13 [14 ± 8] (*n *= 6)	14 [20 ± 17] (*n *= 6)	10 [18 ± 20] (*n *= 6)	6 [8 ± 3] (*n *= 6)
Cerebellum	7 [10 ± 6] (*n *= 6)	8 [13 ± 10] (*n *= 6)	19 [17 ± 6] (*n *= 6)	21 [25 ± 14] (*n *= 6)	9 [12 ± 6] (*n *= 6)	16 [15 ± 3] (*n *= 6)	10 [10 ± 4] (*n *= 6)	14 [14 ± 3] (*n *= 6)
Liver	9 [8 ± 10] (*n *= 6)	12 [18 ± 15] (*n *= 6)	25 [32 ± 25] (*n *= 6)	24 [37 ± 41] (*n *= 6)	8 [8 ± 4] (*n *= 6)	3 [12 ± 15] (*n *= 6)	3 [10 ± 10] (*n *= 6)	6 [12 ± 11] (*n *= 6)
Lung	26 [27 ± 14] (*n *= 6)	23 [23 ± 11] (*n *= 6)	24 [32 ± 22] (*n *= 6)	27 [27 ± 15] (*n *= 6)	35 [39 ± 23] (*n *= 6)	25 [39 ± 31] (*n *= 6)	55 [53 ± 35] (*n *= 6)	36 [47 ± 54] (*n *= 6)
Kidney	6 [36 ± 60] (*n *= 6)	7 [17 ± 23] (*n *= 6)	9 [53 ± 73] (*n *= 6)	9 [28 ± 40] (*n *= 6)	9 [13 ± 13] (*n *= 6)	10 [20 ± 27] (*n *= 6)	8 [12 ± 9] (*n *= 6)	7 [8 ± 5] (*n *= 6)
Bile	5 [6 ± 4] (*n *= 6)	3 [5 ± 4] (*n *= 6)	2 [3 ± 2] (*n *= 6)	6 [6 ± 5] (*n *= 6)	3 [6 ± 7] (*n *= 6)	3 [5 ± 5] (*n *= 6)	3 [3 ± 2] (*n *= 6)	4 [4 ± 3] (*n *= 6)
Duodenum	3 [3 ± 1] (*n *= 6)	4 [13 ± 21] (*n *= 6)	7 [12 ± 13] (*n *= 6)	16 [16 ± 11] (*n *= 6)	6 [7 ± 5] (*n *= 6)	4 [5 ± 4] (*n *= 6)	3 [4 ± 2] (*n *= 6)	7 [7 ± 3] (*n *= 6)
Muscle	6 [6 ± 3] (*n *= 6)	4 [6 ± 6] (*n *= 6)	2 [2 ± 2] (*n *= 6)	3 [3 ± 2] (*n *= 6)	4 [5 ± 4] (*n *= 6)	2 [2 ± 1] (*n *= 6)	1 [2 ± 2] (*n *= 6)	3 [5 ± 4] (*n *= 6)

	RCS-4 Median conc. [Mean conc. ± SD] in ng/mL or ng/g							
	RT				4 °C			
	PMI 0	PMI 1	PMI 2	PMI 3	PMI 0	PMI 1	PMI 2	PMI 3
PB	0.6* [0.6 ± 0.3] (*n *= 6)	0.7* [3± 4] (n=4)	1* [2 ± 1] (*n *= 4)	3* [6 ± 7] (*n *= 4)	0.3 [0.4 ± 0.1] (*n *= 4)	0.3 [0.3 ± 0.04] (*n *= 4)	0.4 [3 ± 4] (*n *= 4)	0.4 [0.6 ± 0.4] (*n *= 4)
CB	1* [2 ± 1] (*n *= 4)	4* [5 ± 3] (*n *= 5)	4* [6 ± 5] (*n *= 5)	7* [15 ± 18] (*n *= 5)	2* (*n *= 2)	4* [5 ± 4] (*n *= 4)	4* [5 ± 5] (*n *= 4)	7* (*n *= 1)
Cerebrum	4 [3 ± 2] (*n *= 6)	4 [4 ± 2] (*n *= 6)	5 [8 ± 6] (*n *= 6)	6 [10 ± 13] (*n *= 6)	4 [6 ± 3] (*n *= 6)	5 [6 ± 5] (*n *= 6)	4 [5 ± 4] (*n *= 6)	7 [6 ± 3] (*n *= 6)
Cerebellum	2 [3 ± 2] (*n *= 6)	2 [3 ± 1] (*n *= 6)	4 [5 ± 2] (*n *= 6)	7 [8 ± 4] (*n *= 6)	4 [4 ± 3] (*n *= 6)	3 [4 ± 3] (*n *= 6)	3 [4 ± 3] (*n *= 6)	7 [7 ± 3] (*n *= 6)
Liver	5 [8 ± 6] (*n *= 6)	10 [12 ± 8] (*n *= 6)	13 [18 ± 16] (*n *= 6)	18 [32 ± 29] (*n *= 6)	6 [5 ± 2] (*n *= 6)	5 [5 ± 2] (*n *= 6)	7 [6 ± 2] (*n *= 6)	9 [9 ± 5] (*n *= 6)
Lung	14 [14 ± 6] (*n *= 6)	21 [23 ± 15] (*n *= 6)	30 [32 ± 25] (*n *= 6)	37 [42 ± 21] (*n *= 6)	23 [26 ± 12] (*n *= 5)	16 [21 ± 15] (*n *= 6)	39 [35 ± 18] (*n *= 6)	27 [35 ± 20] (*n *= 6)
Kidney	5 [8 ± 5] (*n *= 6)	6 [11 ± 11] (*n *= 6)	9 [13 ± 11] (*n *= 6)	8 [14 ± 11] (*n *= 6)	4 [6 ± 4] (*n *= 6)	6 [8 ± 6] (*n *= 6)	7 [12 ± 14] (*n *= 6)	6 [8 ± 7] (*n *= 6)
Bile	7 [8 ± 3] (*n *= 6)	8 [8 ± 4] (*n *= 6)	5 [5 ± 3] (*n *= 6)	6 [6 ± 3] (*n *= 6)	5 [5 ± 4] (6)	4 [5 ± 4] (*n *= 6)	5 [4 ± 2] (*n *= 6)	4 [5 ± 3] (*n *= 6)
Duodenum	6 [6 ± 2] (*n *= 6)	7 [8 ± 4] (*n *= 6)	11 [11 ± 3] (*n *= 6)	13 [18 ± 10] (*n *= 6)	4 [4 ± 3] (*n *= 6)	5 [6 ± 3] (*n *= 6)	3 [5 ± 5] (*n *= 6)	7 [13 ± 13] (*n *= 6)
Muscle	4 [5 ± 2] (*n *= 6)	3 [4 ± 2] (*n *= 6)	5 [6 ± 6] (*n *= 6)	6 [5 ± 2] (*n *= 6)	4 [4 ± 2] (*n *= 6)	5 [6 ± 4] (*n *= 6)	6 [6 ± 4] (*n *= 6)	3 [4 ± 2] (*n *= 6)

	THC Median conc. [Mean conc. ± SD] in ng/mL or ng/g							
	RT				4 °C			
	PMI 0	PMI 1	PMI 2	PMI 3	PMI 0	PMI 1	PMI 2	PMI 3
PB	0.9 (*n *= 2)	1 [3 ± 3] (*n *= 4)	0.7 [0.6 ± 0.2] (*n *= 4)	1 [1 ± 0.8] (*n *= 4)				
CB	0.9 [1 ± 0.9] (*n *= 3)	2* [3 ± 2] (*n *= 4)	0.3 [1 ± 1] (*n *= 4)	1 [1 ± 1] (*n *= 4)				
Cerebrum	9 [12 ± 11] (*n *= 6)	8 [10 ± 7] (*n *= 6)	14 [14 ± 6] (*n *= 6)	23 [31 ± 25] (*n *= 6)	7 [7 ± 6] (*n *= 6)	6 [8 ± 5] (*n *= 6)	8 [10 ± 7] (*n *= 6)	11 [10 ± 6] (*n *= 6)
Cerebellum	9 [9 ± 5] (*n *= 6)	9 [12 ± 8] (*n *= 6)	12 [14 ± 8] (*n *= 6)	13 [18 ± 11] (*n *= 6)	9 [121 ± 7] (*n *= 6)	15 [18 ± 15] (*n *= 6)	12 [11 ± 5] (*n *= 6)	17 [14 ± 9] (*n *= 6)
Liver	37 [36 ± 24] (*n *= 3)	31 [53 ± 38] (*n *= 6)	45 [56 ± 22] (*n *= 6)	73 [78 ± 43] (*n *= 6)	12 [15 ± 9] (*n *= 3)	10 [16 ± 11] (*n *= 6)	19 [16 ± 7] (*n *= 6)	28 [24 ± 14] (*n *= 6)
Lung	10 [11 ± 8] (*n *= 6)	11 [19 ± 20] (*n *= 6)	27 [32 ± 31] (*n *= 6)	15 [18 ± 14] (*n *= 6)	15 [18 ± 13] (*n *= 6)	15 [20 ± 15] (*n *= 6)	14 [19 ± 14] (*n *= 6)	18 [25 ± 22] (*n *= 6)
Kidney	7 [16 ± 18] (*n *= 6)	5 [13 ± 12] (*n *= 6)	9 [28 ± 27] (*n *= 6)	16 [26 ± 22] (*n *= 6)	13 [12 ± 6] (*n *= 5)	7 [8 ± 5] (*n *= 6)	10 [12 ± 8] (*n *= 6)	11 [10 ± 5] (*n *= 6)
Bile	10 [36 ± 69] (*n *= 6)	10 [37 ± 63] (*n *= 6)	17 [24 ± 21] (*n *= 6)	12 [13 ± 4] (*n *= 6)	8 [13 ± 15] (*n *= 6)	8 [15 ± 12] (*n *= 6)	5 [8 ± 7] (*n *= 6)	7 [6 ± 3] (*n *= 6)
Duodenum	48 [78 ± 78] (*n *= 6)	45 [44 ± 16] (*n *= 6)	88 [135 ± 105] (*n *= 6)	82 [129 ± 113] (*n *= 6)	73 [90 ± 65] (*n *= 6)	67 [79 ± 58] (*n *= 6)	27 [79 ± 105] (*n *= 6)	124 [131 ± 116] (*n *= 6)
Muscle	9 [6 ± 4] (*n *= 6)	3 [4 ± 5] (*n *= 6)	3 [3 ± 2] (*n *= 6)	2 [3 ± 2] (*n *= 6)	3 [4 ± 2] (*n *= 6)	5 [4 ± 2] (*n *= 6)	5 [4 ± 2] (*n *= 6)	4 [4 ± 1] (*n *= 6)

A value higher than zero would indicate a concentration increase, a value lower than zero a concentration decrease.

### Statistical tests

A non-parametric Friedman-test (*p* < 0.05) followed by the dunn’s multiple comparison posthoc test was performed to examine the time-dependent concentration changes in the different specimens. A non-parametric Mann–Whitney *U* test (*p* < 0.05) was applied to compare concentrations determined after storage at RT with those determined after storage at 4 °C. Statistics were carried out using GraphPad Prism 5.00 (GraphPad Software, San Diego, CA, USA).

## Results

### Standard addition method

In the current study, the standard addition approach was applied to quantify the drugs and their metabolites in tissues and bile fluid/duodenum content. Regression coefficients (*r*2) for JWH-210, RCS-4 and THC as well as their metabolites ranged between 0.95 and 0.99.

### General remarks

In general, high interindividual differences could be observed in the analyzed PM specimens at the three different PMI under two different storage conditions. The median and mean concentrations (and their standard deviations; SD) of the parent compounds and the metabolites were calculated based on the findings published by Schaefer et al. (2019). They are listed in Tables 1 and 2. As far as the parent compounds are concerned, the analysis of PM specimens revealed highest JWH-210 concentrations in lung, brain, liver and kidney (Table 1). Highest concentrations of RCS-4 were detected in lung, liver and kidney (Table 1). Highest THC concentrations were determined in liver, lung, brain and duodenum content (Table 1). Lowest concentrations of the parent drugs were assessed in PB and CB (Table 1) specimens, respectively. As for the detection of the metabolites, they were primarily determined in tissues/body fluids involved in metabolism or excretion such as liver, lung, bile fluid, and duodenum content (Table 2).

**Table 2:** Median and mean [± standard deviation (SD)] concentrations of HO-JWH-210, HO-RCS-4, RCS-4-COOH, HO-THC and THC-COOH in tissues and body fluids measured at postmortem interval (PMI) 0 according to Schaefer et al. (2019) and PMI 1–3 stored at 4 °C or room temperature (RT); concentrations are approximated (except for PB and CB) and displayed as one decimal non-zero

	HO-JWH-210 Median conc. [Mean conc. ± SD] in ng/mL or ng/g							
	RT				4 °C			
	PMI 0	PMI 1	PMI 2	PMI 3	PMI 0	PMI 1	PMI 2	PMI 3
PB								
CB								
Liver								
Lung	1 [2 ± 2] (*n *= 6)	3 [3 ± 1] (*n *= 6)	1 [2 ± 2] (*n *= 6)	2 [3 ± 2] (*n *= 6)	1 [2 ± 1] (*n *= 4)	1 [1 ± 1] (*n *= 4)	1 [1 ± 1] (*n *= 4)	3 [3 ± 2] (*n *= 4)
Kidney	2 (*n *= 1)				3 (*n *= 1)			
Bile	5 [7 ± 6] (*n *= 6)	8 [11 ± 11] (*n *= 6)	6 [6 ± 5] (*n *= 6)	3 [4 ± 4] (*n *= 6)	7 [8 ± 8] (*n *= 6)	6 [7 ± 7] (*n *= 3)	1 [6 ± 8] (*n *= 3)	2 [5 ± 6] (*n *= 3)
Duodenum	4 [4 ± 3] (*n *= 6)	5 [7 ± 7] (*n *= 6)	6 [6 ± 5] (*n *= 6)	9 [14 ± 14] (*n *= 6)	4 [5 ± 2] (*n *= 6)	4 [4 ± 2] (*n *= 5)	1 [3 ± 3] (*n *= 5)	8 [11 ± 6] (*n *= 5)
Muscle								

	HO-RCS-4 Median conc. [Mean conc. ± SD] in ng/mL or ng/g							
	RT				4 °C			
	PMI 0	PMI 1	PMI 2	PMI 3	PMI 0	PMI 1	PMI 2	PMI 3
PB		0.8 [0.8 ± 0.7] (*n *= 3)	0.7 [2 ± 3] (*n *= 4)	1 [3 ± 3] (*n *= 3)				
CB		2 [6 ± 6] (*n *= 4)	8 [7 ± 4] (*n *= 4)	1 [0.8 ± 0.5] (*n *= 4)				
Liver	4 [6 ± 4] (*n *= 6)	6 [7 ± 5] (*n *= 6)	7 [27 ± 50] (*n *= 6)	9 [12 ± 6] (*n *= 6)	5 [5 ± 3] (*n *= 6)	8 [8 ± 5] (*n *= 6)	5 [6 ± 3] (*n *= 6)	6 [11 ± 12] (*n *= 6)
Lung	1 [1 ± 1] (*n *= 5)	2 [2 ± 2] (*n *= 6)	5 [5 ± 3] (*n *= 6)	3 [2 ± 1] (*n *= 6)	1 [1 ± 1] (*n *= 4)	3 [3 ± 2] (*n *= 6)	1 [2 ± 2] (*n *= 6)	2 [2 ± 1] (*n *= 6)
Kidney	4 [6 ± 4] (*n *= 6)	4 [6 ± 4] (*n *= 6)	5 [9 ± 4] (*n *= 6)	7 [7 ± 5] (*n *= 6)	4 [7 ± 6] (*n *= 6)	6 [18 ± 26] (*n *= 6)	9 [6 ± 6] (*n *= 6)	8 [7 ± 6] (*n *= 6)
Bile	91 [188 ± 239] (*n *= 6)	135 [198 ± 139] (*n *= 6)	132 [164 ± 108] (*n *= 6)	110 [100 ± 67] (*n *= 6)	102 [128 ± 93] (*n *= 6)	94 [90 ± 25] (*n *= 6)	99 [101 ± 40] (*n *= 6)	61 [69 ± 30] (*n *= 6)
Duodenum	85 [116 ± 81] (*n *= 6)	40 [61 ± 55] (*n *= 6)	123 [131 ± 76] (*n *= 6)	100 [253 ± 409] (*n *= 6)	92 [92 ± 44] (*n *= 6)	71 [87 ± 77] (*n *= 6)	55 [67 ± 59] (*n *= 6)	34 [97 ± 103] (*n *= 6)
Muscle	2 [2 ± 1] (*n *= 3)	2 [2 ± 1] (*n *= 6)	1 [2 ± 1] (*n *= 6)	1 [2 ± 2] (*n *= 6)	1 (*n *= 2)	2 (*n *= 2)	1 (*n *= 2)	1 (*n *= 2)

	RCS-4-COOH Median conc. [Mean conc. ± SD] in ng/mL or ng/g							
	RT				4 °C			
	PMI 0	PMI 1	PMI 2	PMI 3	PMI 0	PMI 1	PMI 2	PMI 3
PB								
CB								
Liver								
Lung								
Kidney								
Bile	142 [154 ± 48] (*n *= 6)	205 [236 ± 185] (*n *= 6)	170 [179 ± 113] (*n *= 6)	140 [114 ± 71] (*n *= 6)	96 [101 ± 30] (*n *= 6)	165 [152 ± 43] (*n *= 6)	147 [151 ± 14] (*n *= 6)	81 [85 ± 65] (*n *= 6)
Duodenum	134 [157 ± 135] (*n *= 5)	52 [87 ± 61] (*n *= 6)	118 [132 ± 43] (*n *= 6)	138 [178 ± 133] (*n *= 6)	57 [103 ± 108] (*n *= 6)	41 [52 ± 43] (*n *= 6)	12 [15 ± 9] (*n *= 6)	30 [42 ± 28] (*n *= 6)
Muscle								

	HO-THC Median conc. [Mean conc. ± SD] in ng/mL or ng/g							
	RT				4 °C			
	PMI 0	PMI 1	PMI 2	PMI 3	PMI 0	PMI 1	PMI 2	PMI 3
PB								
CB								
Liver								
Lung								
Kidney								
Bile	241 (*n *= 2)	229 (*n *= 2)	232 (*n *= 2)	360 (*n *= 2)	82 [114 ± 118] (*n *= 4)	68 [68 ± 17] (*n *= 6)	157 [157 ± 143] (*n *= 6)	118 [119 ± 8] (*n *= 6)
Duodenum								
Muscle								

	THC-COOH Median conc. [Mean conc. ± SD] in ng/mL or ng/g							
	RT				4 °C			
	PMI 0	PMI 1	PMI 2	PMI 3	PMI 0	PMI 1	PMI 2	PMI 3
PB								
CB								
Liver								
Lung	100 (*n *= 1)	42 (*n *= 1)	57 (*n *= 1)	254 (*n *= 1)	36 [28 ± 14] (*n *= 3)	26 [22 ± 9] (*n *= 3)	41 [35 ± 22] (*n *= 3)	24 [19 ± 14] (*n *= 3)
Kidney	5 [21 ± 31] (*n *= 3)	16 [55 ± 79] (*n *= 3)	13 [19 ± 15] (*n *= 3)	17 [17 ± 8] (*n *= 3)	13 (*n *= 1)	10 (*n *= 1)	5 (*n *= 2)	8 (*n *= 2)
Bile	32 [105 ± 137] (*n *= 3)	78 [68 ± 50] (*n *= 3)	51 [45 ± 22] (*n *= 3)	117 (*n *= 1)	56 [69 ± 51] (*n *= 5)	27 [27 ± 9] (*n *= 4)	43 [50 ± 14] (*n *= 4)	50 [86 ± 99] (*n *= 4)
Duodenum	102 (*n *= 1)	91 (*n *= 1)	55 (*n *= 1)	10 (*n *= 1)	65 (*n *= 2)	68 (*n *= 2)	88 (*n *= 2)	270 (*n *= 2)
Muscle								

*PB* peripheral blood, *CB* central blood

### JWH-210 and metabolites

#### Storage at RT

**Fig. 1:**
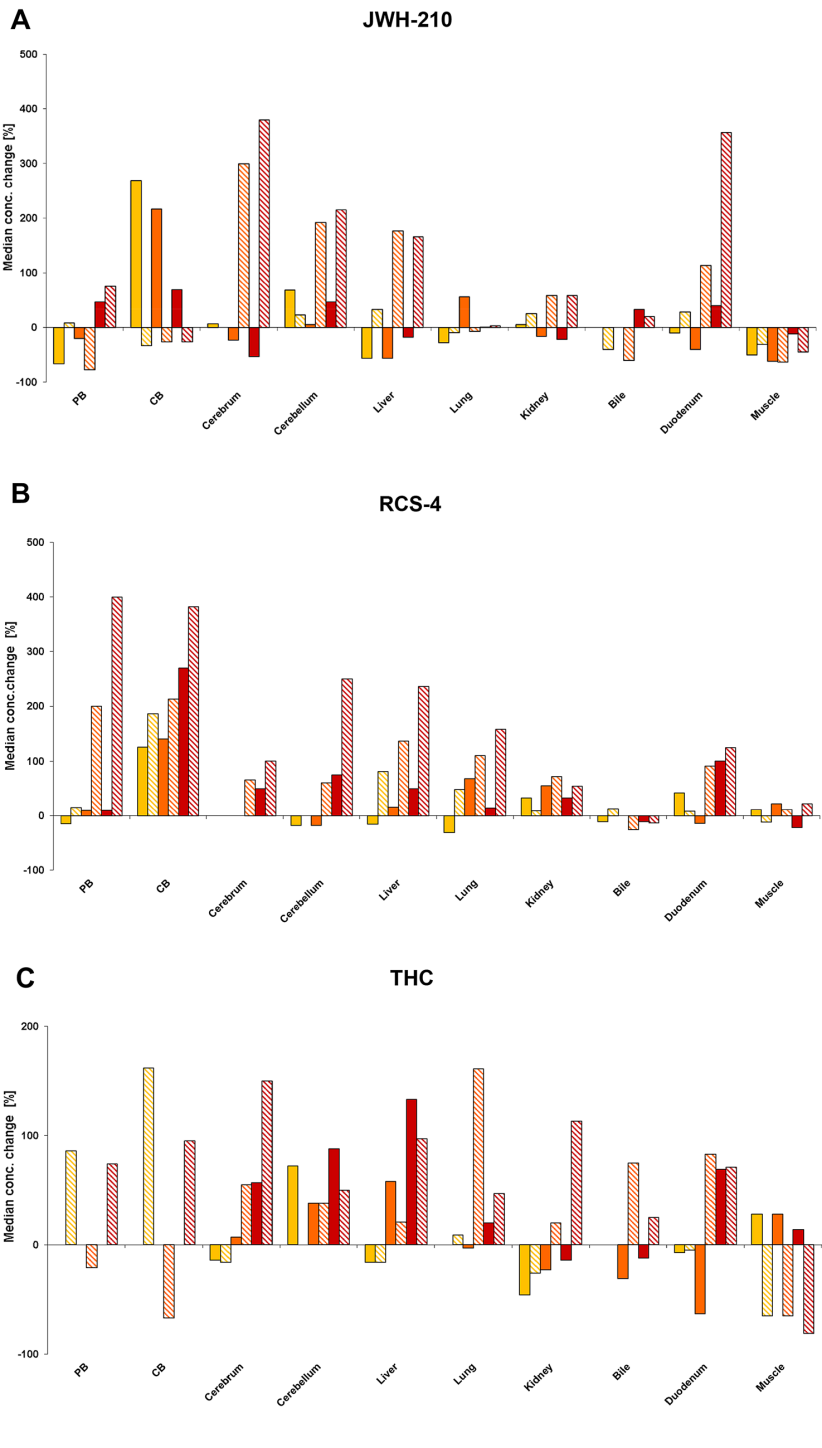
Time- and temperature-dependent postmortem concentration changes of **a** JWH-210, **b** RCS-4 and **c** THC.  PMI 1 (4 °C),  PMI 1 (RT);  PMI 2 (4 °C);  PMI 2 (RT);  PMI 3 (4 °C);  PMI 3 (RT); in pig tissues and body fluids following pulmonary administration of a 200 µg/kg body weight dose each. Concentrations are displayed as the median concentration change compared to concentrations calculated at PMI 0 (Schaefer et al. 2019); *PB* peripheral blood, *CB* central blood, *RT* room temperature (color figure online)

JWH-210 was reliably quantified in every PM specimen of pigs stored at RT except for PB specimens, with JWH-210 being detected in only four pigs (Table 1). In CB, lung, bile and muscle tissue only slight concentration changes were measured (Fig. 1a; Table 1). Median concentrations in brain, liver, kidney, and duodenum increased continuously PM. Concerning PB, median concentrations increased from PMI 0 to 1, decreased from PMI 1 to PMI 2 and increased to PMI 3 and (Fig. 1a; Table 1).

As far as HO-JWH-210 is concerned, detection in the PM specimens was successful in lung, bile, and duodenum (*n* = 6 each; see Table 2). Comparing the median concentrations assessed at PMI 1–3 with those determined immediately after death (Table 2), no relevant change was observed in lung tissue. In bile, concentrations increased until 24 h after death and decreased again. Mean concentrations of HO-JWH-210 in bile were significantly (*p* < 0.05) higher at PMI 1 as compared to PMI 3. In duodenum content, concentrations slightly increased from PMI 0 (Schaefer et al. 2019) to PMI 3.

No JWH-210-COOH could be detected in the PM specimens.

#### Storage at 4 °C

JWH-210 could be detected in every PM specimen of pigs stored at 4 °C except for PB and CB (see Table 1). In those specimens, a detection could only be achieved in four out of six pigs, respectively. Compared to PMI 0 (see Table 1), median concentrations of JWH-210 at PMI 1 were higher in CB, brain and kidney and lower in PB, liver, lung, duodenum, and muscle tissue (Fig. 1a; Table 1). On the contrary, concentrations decreased from PMI 1 to 2 in CB, brain, kidney, and muscle, while concentrations in PB and lung increased (Fig. 1a; Table 1). At PMI 3, median concentrations were slightly higher in blood, cerebellum, lung, bile and duodenum, and slightly lower in cerebrum, liver, kidney and muscle tissue as compared to median concentrations measured at PMI 0 (Fig. 1a; Table 1).

With regard to the main metabolite HO-JWH-210, it was reliably detected and quantified at PMI 1–3 in lung (*n* = 4), bile (*n* = 3), and duodenum (*n* = 5; Table 2). In lung and duodenum, the median concentrations calculated at PMI 3 were higher as compared to PMI 0 (Table 2). Mean concentrations in duodenum content calculated at PMI 1 were significantly (*p* < 0.05) lower as compared to those measured at PMI 3. In bile fluid, concentrations declined continuously over time (Table 2). No JWH-210-COOH was detected in the PM specimens.

### RCS-4 and metabolites

#### Storage at RT

RCS-4 could be determined in every PM specimen of pigs stored at RT except for PB (*n* = 4) and CB (*n* = 5, see Table 1). Compared to median concentrations at PMI 0 (Table 1), median PM concentrations increased continuously in every specimen except for bile fluid and muscle tissue (Fig. 1b; Table 1). Only little changes (increases and decreases) were observed in those specimens (Fig. 1b; Table 1). In PB and CB, the highest concentration increases were observed (Fig. 1b; Table 1).

Concerning HO-RCS-4, this metabolite could be determined in every PM specimen except for PB, CB and brain (*n* = 0, Table 2). In PB specimens of three pigs, detection was achieved over two days, in one pig only specimens collected at PMI 2 and 3 contained HO-RCS-4, and in one pig it was only detected 72 h after death. In those PB specimens tested positive for HO-RCS-4, a concentration increase was assessed from PMI 1 to PMI 3 (Table 2). As far as CB is concerned, HO-RCS-4 was detected in four pigs showing concentration increases until 48 h PM and decreases from 48 to 72 h PM (Table 2). Besides duodenum content, median concentrations were equal or higher during storage at ambient temperature for 3 days in comparison to median concentrations at PMI 0 (Table 2). In duodenum content, PM concentrations decreased from PMI 0 to PMI 1 and increased again (Table 2).

RCS-4-COOH was only detected in bile fluid and duodenum content (Table 2). Median concentrations in bile increased from PMI 0 to PMI 1 and decreased again in the following two days. Mean concentrations were significantly (*p* < 0.05) higher at PMI 1 as compared to PMI 2. On the contrary, median concentrations in duodenum decreased from PMI 0 to PMI 1 and increased again in the following two days (Table 2).

#### Storage at 4 °C

Besides PB and CB specimens (*n* = 4 each; *n* = 1: RCS-4 at PMI 3, see Table 1), RCS-4 was reliably quantified in every PM specimen of pigs stored at 4 °C. Only slight changes were observed in PB, brain, bile fluid, and muscle. In most of the remaining specimens median concentrations calculated at PMI 1–3 were higher as compared to those determined at PMI 0 with CB showing the most considerable differences (Fig. 1b; Table 1).

As can be seen in Table 2, HO-RCS-4 could be determined in every PM specimen except for PB (*n* = 0), CB (*n* = 0), brain (*n* = 0), and muscle (*n* = 2). Median concentrations calculated in specimens stored at 4 °C for 3 days were slightly lower or higher in comparison with concentrations at PMI 0 (Table 2). Overall decreases were observed in bile and duodenum with mean concentrations showing great SD.

RCS-4-COOH was only detected in bile fluid and duodenum content (Table 2). Median concentrations in bile fluid increased from PMI 0 to PMI 1 and decreased again until PMI 3. Concentrations in duodenum decreased until PMI 2 and increased again from PMI 2 to PMI 3. A statistical significant difference (*p* < 0.05) could be observed at PMI 2 as compared to PMI 0 (Table 2).

### THC and metabolites

#### Storage at RT

Concerning the PM PB and CB specimens, THC could only be detected in four pigs. In those specimens, concentrations were higher at PMI 1 and 3 and lower at PMI 2 as compared to those measured at PMI 0 (Fig. 1c; Table 1). Overall, the median concentrations determined at PMI 1–3 were only slightly lower or higher (< 200%) than those quantified at PMI 0 (Fig. 1c; Table 1). In brain, liver, lung, and kidney median concentrations showed a tendency to increase from PMI 2 to PMI 3 (Fig. 1c; Table 1). At PMI 3, significantly higher (*p* < 0.05) mean concentrations were determined in cerebrum, cerebellum, and duodenum as compared to PMI 0 or 1, respectively (Table 1).

HO-THC was only detectable in bile fluid samples of two pigs. No considerable changes were observed comparing the median concentrations at PMI 0 with those at PMI 1–2 (Table 2). At PMI 3 median concentrations were higher as compared to those measured at PMI 0 (Table 2).

In terms of the detectability of THC-COOH in the PM specimens, it was determined in the lung tissue and duodenum content (PMI 1–3) of only one pig with lung showing a slight decrease from PMI 0 to PMI 2 and an increase from PMI 2 to PMI 3 and duodenum content revealing a time-dependent decline of the concentration (Table 2). In kidney and bile fluid, THC-COOH was detected in three pigs (and in bile at PMI 3 in one pig) showing tendencies to increase (Table 2).

#### Storage at 4 °C

THC could be determined in every PM specimen stored at 4 °C except for PB and CB (*n* = 0 each) specimens (Table 1). In terms of the remaining tissues and body fluids median concentrations changed only slightly (< 200%) with increasing PMI as compared to concentrations determined at PMI 0 (Fig. 1c; Table 1). Median concentration in brain and liver tissue tended to increase while concentrations in kidney appeared to decrease (Fig. 1c; Table 1). Concentrations determined in duodenum decreased from PMI 0 to PMI 2 and increased again. After 72 h they were higher as compared to those measured at PMI 0 (Table 1).

HO-THC could only be determined in PM bile fluid specimens showing a tendency to increase (Table 2).

Regarding THC-COOH, this metabolite could be detected in lung (*n* = 3) and kidney (*n* = 1–2) tissue as well as bile fluid (*n* = 4) and duodenum content (*n* = 2; Table 2). Comparing the median PM with the median concentrations at PMI 0 (Table 2), only slight differences could be observed except for duodenum content (Table 2). Concentrations in kidney were lower as compared to those found at PMI 0 (Table 2). Concentrations in lung decreased from PMI 0 to 1, increased from PMI 1 to 2 and decreased again (Table 2). Concentrations in bile decreased from PMI 0 to PMI 1 and increased again, whilst concentrations in duodenum content increased over time (Table 2).

### Comparison of concentrations at different storage conditions

Comparison of the two storage temperatures revealed significantly (*p* < 0.05) different mean concentrations in CB (JWH-210), PB (RCS-4, THC), cerebrum (JWH-210), liver (RCS-4), bile (THC), and duodenum (JWH-210, Table 1).

## Discussion

### Dosage

Regarding the dosage, the total doses of 8.1–9.9 mg of JWH-210, RCS-4, and THC administered in the current study, are comparable with SC doses administered in the few systematic controlled animal studies or human self-experiments (Castaneto et al. 2015) as well as THC doses used in human studies with inhalative consumption (Desrosiers et al. 2014; Hazekamp et al. 2006).

### Standard addition method

In our previous study (Schaefer et al. 2019) dealing with the concentrations of the drugs in tissues at the time of death as well as in the present study focusing on the changes of the concentrations during a PMI of 3 days, the standard addition approach was applied for quantification of the drugs in tissues and bile fluid/duodenum content. The concentrations of the calibrators were determined according to a rough semiquantitative estimation of the amount found in initial analyses. The calibrator concentrations were accordingly adjusted. The calibration curves were regarded to be linear with *r*2 > 0.95.

The standard addition approach is more laborious as compared to the conventional method validation. However, it has the advantage of minimizing matrix effects (Jickells and Negrusz 2008), because each calibration curve is matrix-matched. This is of particular importance in PM toxicology, because matrix effects can be challenging due to purification of specimens.

A common validation procedure requires the assessment of several parameters using blank matrix from different individuals. Though, in case of PM specimens it is questionable, whether this would lead to representative results, as the interindividual biological variances of the same matrix specimens are very high. Thus, national and international guidelines recommend the standard addition approach for quantification of drugs in PM specimens (GTFCh 2018; Jickells and Negrusz 2008; Peters et al. 2007; Skopp 2010; SOFT/AAFS 2006). Following this recommendation, this procedure has frequently been applied in quantitative PM studies on the tissue distribution of drugs (Hasegawa et al. 2014; Mochizuki et al. 2019; Schaefer et al. 2017b; Siek and Dunn 1993). In particular in case reports of human fatalities the standard addition method has been used for PM analysis indicating that this is the prevailing analytical procedure in routine PM examination.

### PM distribution

The perimortem distribution of the drugs and their metabolites in tissues and body fluids has already been described elsewhere (Schaefer et al. 2019). In brief, highest concentrations of JWH-210 were found in lung, kidney, and brain. Highest concentrations of RCS-4 were determined in the lung, and highest THC concentrations in liver, bile fluid, and duodenum content. Lowest concentrations of the parent compounds were detected in PB and CB specimens, respectively (Schaefer et al. 2019). As far as the detection of metabolites is concerned, they were only determined in tissues/body fluids involved in metabolism or excretion such as liver, lung, bile fluid, and duodenum content. On the contrary, no metabolites could be detected in PB and CB (Schaefer et al. 2019). The PM distribution of the drugs and their metabolites assessed in the current study is comparable to the perimortem findings (Tables 1, 2).

In respect of the SC JWH-210 and RCS-4, no further data have been published regarding their PM tissue distribution or possible potential to undergo PMR. Scientific data on the distribution of SC are very sparse. Indeed, many fatal intoxications have been reported, but in these authentic cases (as it is usual the problem with single case reports) time of intake and dose or the PM interval were unknown (Castaneto et al. 2015; Kraemer et al. 2019; Meyer 2016). Concerning PM data of THC, Brunet et al. studied the time-dependent PMR of THC in pigs following i.v. administration of a 200 µg/kg BW dose (Brunet et al. 2010). Their findings will be discussed below.

### PM concentration changes and possible explanations

Postmortem time-dependent concentration changes of the cannabinoids were observed throughout the tested specimens. As expected, the changes were more considerable during storage at RT (Fig. 1a–c; Table 1). However, statistical significance of the observed concentration changes should generally be interpreted with caution, because the interindividual differences commonly encountered in PM cases are very high. Particularly analyzing tissues (Brunet et al. 2010; Nagasawa et al. 2016), but also determining concentrations in PM blood specimens (Saar et al. 2012) revealed comparably high interindividual deviations, even if the analytical method was fully validated. This correlation may indicate that this high variance is attributable to the enormous biological variability of PM degraded tissues rather than to inaccuracy oft the analytical procedure.

In general, several parameters can be taken into consideration to estimate the probability of a substance undergoing PMR. One marker could be a central-to-peripheral blood concentration ratio (*C*/*P*-ratio) of > 1 (Han et al. 2012). As already discussed in our previous study (Schaefer et al. 2019), this aspect implicates a PMR-potential especially of RCS-4. The data of the present study further reinforce this assumption (Table 1). Considering the large volumes of distribution of 4.9 (JWH-210), 4.1 (THC), and 15 L/kg^0.75^ (RCS-4) (Schaefer et al. 2018a, b), which is another predictor for a drug underlying PMR (Hilberg et al. 1999), again a PMR-potential particularly of RCS-4 can be suggested. A further marker to assess PMR of a drug is the liver-to-peripheral blood ratio (*L*/*P*-ratio) (McIntyre 2014). McIntyre et al. published data prompting the conclusion that an *L*/*P*-ratio of less than 5 might implicate little or no PMR, while an *L*/*P*-ratio greater than 20–30 would suggest a high PMR-potential (McIntyre 2014). In terms of the *L*/*P*-ratios calculated with the previous (Schaefer et al. 2019) as well as the current data (see Table 1), most of the ratios are greater than 5, additionally confirming a PMR-potential.

Furthermore, a various number of issues has to be kept in mind in the attempt to explaining PM alterations of a drugs concentrations. The most common interpretation of the term PMR refers to the distribution of a drug from a drug reservoir (organ or tissue in which a drug is stored in high concentrations) to surrounding organs or tissues (Pélissier-Alicot et al. 2003). However, further issues have to be taken into consideration. Changes in drug concentrations might be additionally caused by physiological processes expiring during agony or PM. One example is the loss of adenosine triphosphate production leading e.g. to glycogen depletion and acidification of the cell (Pélissier-Alicot et al. 2003). As a result, basic drugs are trapped and accumulated into the cell. Another example is the PM blood coagulation. Coagulated blood includes a high number of red blood cells, leading to variations of hematocrit. This issue might be of importance when interpreting PM blood concentrations, because some drugs exhibit an unequal distribution between erythrocytes and serum/plasma (Kennedy 2010; Pélissier-Alicot et al. 2003). In this context, the hypostasis, characterized by the sedimentation of blood and plasma to lower parts of the body, leading to changes in the percentage of the red blood cells by volume, can also affect drug concentrations (Pélissier-Alicot et al. 2003). Last not least, putrefactive processes can further cause PM neogenesis or degradation (e.g. hydrolysis of glucuronides) of substances (Kennedy 2010; Pélissier-Alicot et al. 2003).

However, PMR is not only dependent on agonal and PM physiological changes, but also on the physicochemical and pharmacokinetic properties of a substance. For example, some drugs are highly protein bound. Following, drug concentrations can increase after death due to protein breakdown (Pélissier-Alicot et al. 2003).

To discuss possible explanations for the observed median changes, one has to focus on the absolute concentrations. The median concentrations of JWH-210 in PB and CB were below 1 ng/mL in most of the specimens suggesting the conclusion that the discrepancies might be explained by analytical and interindividual variations. Furthermore, PB specimens derived from different veins, which could also have led to various concentrations. The slight differences of the median concentrations of the remaining specimens stored at 4 °C and RT (less than 100% concentration change) can also be considered as interindividual as well as analytical variations (Fig. 1a; Table 1). The higher concentration changes determined in liver might be caused by an inhomogeneous distribution within this organ combined with a sampling from different sites. A varying accumulation in different brain areas, again associated with non-standardized sampling, might also be an explanation for the increased concentrations measured in brain specimens stored at RT (Fig. 1a; Table 1).

One further issue is noteworthy with regard to possible PM processes. Although being highest at PMI 0 in lung tissue (Schaefer et al. 2019), JWH-210 seems to be stored unalteredly in this organ (Fig. 1a; Table 1). Surprisingly, PMR from this drug reservoir might not be relevant concerning this drug, as indicated by the analytical findings in this tissue.

As for HO-JWH-210 the median concentrations in lung tissue, bile fluid and duodenum content (Table 2), only little differences were observed, again possibly being explained by interindividual and analytical variation.

Again, concentration changes of RCS-4 less than 100% might be attributable to interindividual and analytical variations (Fig. 1b). As for the more obvious changes in PB and CB, a PM release of RCS-4 from red blood cells and hence change in the hematocrit might be a plausible explanation. A previous study of the authors provided data on the distribution of RCS-4 in serum and whole blood samples, leading to the conclusion that RCS-4 might accumulate in red blood cells (Schaefer et al. 2015). Besides a possible PMR from bile fluid to liver and lung tissue, a PM release from red blood cells might be the most important reason for the constant increase of concentrations in these highly vascularized organs (Fig. 1b; Table 1). The high increase in cerebellum from PMI 2 to PMI 3 might be a result of redistribution from cerebrum (Fig. 1b).

Regarding HO-RCS-4, the concentration changes in tissues stored at 4 °C can be considered as minor (Table 2). Storing at RT revealed higher concentration changes, particularly in PB and CB (Table 2). In those specimens, HO-RCS-4 could be detected at PMI 1–3, while no HO-RCS-4 was found in the specimens analyzed at PMI 0 (Table 2) (Schaefer et al. 2019). Besides a redistribution from neighboring tissues, one explanation for these findings might be a PM release of free HO-RCS-4 from its glucuronide due to PM hydrolysis mechanisms. A further remarkable result is that the most considerable increases, especially in blood (and duodenum content) occurred from PMI 1 to PMI 2 whilst significant decreases were observed from PMI 2 to 3 (Table 2).

RCS-4-COOH could only be detected in bile fluid and duodenum content. The increasing concentrations in bile fluid and decreasing concentrations in duodenum content (especially from PMI 0 to PMI 1) might be corresponding to PMR from one tissue to the other (Table 2).

Regarding THC, only minor concentration changes (below 200%) were observed at both storage conditions (Fig. 1c; Table 1). In terms of PB and CB, most of the values laid near the limit of detection (0.5 ng/mL) or lower limit of quantification (2.0 ng/mL (Schaefer et al. 2015)) leading to high analytical variations. However, increases in PB could have resulted from a distribution out of peripheral muscle tissue (Fig. 1c; Table 1). In addition, a sampling from different peripheral veins should be considered. The increases in cerebrum at RT might be a result of PMR from brain areas with higher THC concentrations, as we did not differentiate between the different areas. An unequal distribution of THC in brain has already been described in literature (Brunet et al. 2010). Brunet et al. also observed a marked time-dependent increase of concentrations in brain (Brunet et al. 2010). In general, the results of their study are comparable with our findings. Discrepancies might be at least in parts explained by the different number of animals involved and a shorter PMI.

In terms of lung and liver tissue, a possible inhomogeneous distribution and following variation in concentrations can be assumed, as we did not differentiate between different lobes. This phenomenon has been described in literature (Brunet et al. 2010). The increase of THC concentrations in kidney tissue stored at RT might be attributable to PMR from perirenal adipose tissue, however, the analysis of this tissue was not subject of the current study.

Regarding the metabolites, too few data could be obtained to discuss possible mechanisms.

## Limitations of this study

First of all, it should be noted that the abdominal cavity had to be opened to obtain the PM specimens. This could have resulted in a more aerobic environment and a faster and more pronounced occurrence of microorganisms as well as changes of the microbiome and thus a more distinct putrefication (at least during storage at RT). Furthermore, during the sampling procedure it was not differentiated between different areas of the organs. As drugs might be distributed inhomogenously throughout an organ, this issue should be considered in further studies. At last, the distribution in adipose tissue was not assessed, as it is part of a further study.

## Conclusion

Postmortem distribution patterns as well as time- and temperature-dependent concentration changes of the two SC JWH-210 and RCS-4 and THC following pulmonary administration were investigated in pigs. Lung, liver, kidney and bile fluid/duodenum content are suitable specimens for PM analysis, as comparably high concentrations were observed in those tissues. Furthermore, brain might be an alternative specimen. As far as the metabolites are concerned, HO-RCS-4 was the most prevalent metabolite. However, time-dependent concentrations changes were observed throughout the tested specimens, particularly with regard to RCS-4 and HO-RCS-4. Those changes were more considerable during storage at RT leading to the conclusion that RCS-4 is most prone to PMR.
